# Platform trials—an emerging methodology for perioperative medicine: a narrative review

**DOI:** 10.1186/s13741-025-00543-7

**Published:** 2025-07-04

**Authors:** Tom E. F. Abbott, Sarah-Louise Watson, Salma Begum, Priyanthi Dias, Joanne S. Haviland, James Glasbey, Lawani Ismail, Sharon Love, Rupert M. Pearse

**Affiliations:** 1 Critical Care and Perioperative Medicine Research Group, William Harvey Research Institute, Queen Mary University of London, London, UK; 2 Peter’s Hospital, Ashford and St Peter’s Hospitals NHS Foundation Trust, Chertsey, UK; 3 Pragmatic Clinical Trials Unit, Wolfson Institute of Population Health, Mary University of London, London, Queen UK; 4 NIHR Global Health Research Unit On Global Surgery, Birmingham, UK; 5 Faculty of Health Science (FSS), University of Abomey-Calavi, Contonou, Benin; 6 MRC Clinical Trials Unit at UCL, University College London, London, UK; 7 Department of Anaesthesia & Perioperative Medicine , Royal London Hospital, London, E1 1 FR UK

**Keywords:** Anaesthesia, Perioperative, Surgery, Trials, Platform trials

## Abstract

The traditional model for testing new treatments, before widespread usage in clinical practice, is the parallel group randomised trial. However, these are often inefficient, time-consuming and expensive, which can be barriers to the timely improvement of clinical care. This is a particular issue for anaesthesia and perioperative medicine where funding for large clinical trials is often scarce. Platform trials are an emerging methodology for testing new interventions, which offer benefits over the traditional parallel group paradigm. Platform trials have the ability to test multiple interventions at the same time, and to add or remove interventions during the course of the programme without undermining the validity or integrity of the trial findings. They are most often structured around a master protocol, which describes the core methods and research governance processes, with each intervention described in either a sub-section or appendix to the master protocol. The principal benefit to researchers and to research funders is that, unlike the sequential parallel group trial model, platform trials can use the same research infrastructure (e.g. database, standard operating procedures etc.) to answer multiple research questions, which is much more time and cost effective. The benefits of platform trials can be further enhanced with the use of adaptive designs or by sharing control patients, for example, by using a multi-arm multi-stage design. Perioperative medicine, anaesthesia and surgery are ideally placed to benefit from platform trials.

## Background

Five million people undergo surgery in the UK every year (Abbott et al. 2017). Almost one in six patients develop postoperative medical complications, for example, infections, respiratory failure and myocardial infarctions, representing over 800,000 people annually (Abbott et al. 2018; Abbott et al. 2018; Hui et al. 2022; Dias et al. 2022; group Pt 2021; group ISOS 2016; Abbott et al. 2021; Ackland et al. 2020; Fowler et al. 2022). The risk of complications is increased among older patients and those with chronic conditions or impaired functional capacity (Fowler et al. 2019; Fowler et al. 2022; Wijeysundera et al. 2018). Postoperative complications substantially reduce, and often neutralise, the benefits of surgery for individual patients, decreasing both quality and quantity of life (Fowler et al. 2022; Ladha et al. 2021; Jerath et al. 2019). Although the absolute risk of death within one year after surgery remains small (3.3%), the overall national health impact (~ 165,000 deaths) is significant because the surgical population is very large (Fowler et al. 2022). Over a typical lifetime, 60% of the population will undergo some form of surgery (Watson et al. 2024). Thus, even small improvements in postoperative survival could save many lives.

The majority of perioperative clinical trial questions are about the efficacy or effectiveness of simple drug or device interventions, which require large patient samples due to small effect sizes. Almost all of these trials utilise a two-group randomised design to answer a single research question. However, this approach is time-consuming and resource intensive, such that a typical perioperative clinical trial takes five years to conduct and two years to publish, with most treatments requiring several trials to generate definitive evidence (Hanney et al. 2015; Morris et al. 2011). For drug interventions, the timeline from development of the drug to introduction into clinical practice can be as long as 17 years (Morris et al. 2011). The current model of clinical trials may negatively impact the participant diversity within the trial sample, which limits the external validity of the results within a system that is poorly optimised for both patients and researchers.

Platform trials offer a new and evolving alternative to the traditional model of parallel group randomised trials, by providing the facility to test multiple new treatments within a single unified research governance infrastructure. This model has been successfully used in other fields and is increasingly becoming the default clinical trial model in some clinical research areas. In this review article, we examine the use of platform trials and how they might apply to perioperative research.

### Parallel group trials

The traditional, and most commonly used, model for clinical trials is the parallel group trial, where a group of patients receiving an intervention are compared to a group of patients receiving a control, which is often either standard clinical care or a placebo in the case of a drug trial. The gold standard approach requires random allocation of patients to either the intervention or control group, such that, on balance, the only difference between groups will be the presence or absence of the intervention. This allows the investigator to estimate the effect of the intervention on an outcome measured in both groups, without the influence of other confounding factors that limit the internal validity of non-randomised studies (Nair 2019). There are various adaptions to parallel group trials, including multi-arm trials where several interventions are compared to a control group; cross-over trials, where all patients receive both intervention and control treatments after a suitable washout period; and factorial designs, where patients receive combinations of interventions. The central philosophy of clinical trials (and clinical research more broadly) is that a clinical trial (or study) is set up to answer a defined research question, or set of questions, after which the clinical trial ends, the results are published, and a new trial is set up to answer the next research question.

### Limitations of the parallel group trial model

This traditional model of sequential parallel group trials has several limitations. Firstly, each clinical trial requires its own research governance infrastructure, which includes competitive grant funding, a trial protocol, a steering committee, a team of people to manage the trial (often nested within a Clinical Trials Unit) including trial managers, data managers and statisticians, review by the trial Sponsor, research ethics committee and regulator (in the UK this is either the MHRA or HRA) and a trial database to manage the research data. For clinical trials within a single clinical specialty like anaesthesia and perioperative medicine, much of the research governance infrastructure from the protocol to the case report form is very similar between clinical trials, within only small differences accounting for the intervention, specific patient group and regulatory requirements. Therefore, much of the central infrastructure for clinical trials is duplicated each time a new trial is established. Secondly, when a multi-centre trial is set up at a participating site or hospital, each new centre will go through a stereotyped process of reviewing the trial protocol and documentation, confirming capacity to deliver the trial, training staff in the trial procedures and starting to recruit patients. This process is repeated for each new trial, and the identification and opening of new sites often account for considerable lag in patient recruitment at the start of a multi-centre clinical trial. Consequently, the process of conducting sequential parallel group trials is resource intensive, aimlessly repetitive and time-consuming, with each cycle taking between four and eight years for a typical perioperative trial and up to twelve years for a drug trial (Paul et al. 2010; Bhatt and Mehta 2016).

### Participant diversity in clinical trials

There is an increasing recognition that clinical trials are often not representative of the general patient population, which limits their external validity and the generalisability of the results. In many trials, diversity characteristics, like sex and gender, ethnicity, socioeconomic status and disability are inadequately measured and reported. For example, a review of clinical trials for stroke reported that 37% of 115 trials published between 2010 and 2020 reported sex, which varied between journals and geographic regions; during the COVID-19 pandemic, 98% of trials did not report ethnicity; and a review of clinical trials published in general medical journals in 2019 found that 51% of 358 trials reported race and 12% reported socioeconomic status (Pudar et al. 2021; Pan et al. 2020; Alegria et al. 2015). Where patient characteristics are reported, in many cases, the samples are unbalanced according to one or more characteristics. This is most obvious in speciality areas with a large number of clinical trials, for example, cardiovascular medicine. In a recent review of trials for ischaemic heart disease, over half (53%) of over 800 trials excluded patients aged over 75 or 80 years making the results not applicable to elderly patients, while among heart failure trials, women made up only 25% of the trial samples, despite representing 42–55% of patients with heart failure (Filbey et al. 2023; Ødegaard et al. 2020; Störk et al. 2017). A review of 2485 trials of surgical patients found that 33% reported ethnicity or race, while a review of 51 trials in orthopaedic surgery found that 98% reported age, 96% reported sex, and 37% reported ethnicity (Issa et al. 2023). Low- and middle-income countries are often poorly represented in large multi-centre clinical trials, with only 3% of cardiovascular trials including patients from Africa and less than 25% of trials including patients from outside of Europe and North America (Filbey et al. 2023).

## Complex clinical trials and master protocol studies

Within the constraints of a traditional parallel group trial, it is often not possible (or very difficult) to change the intervention during the course of the study. In fact, from the perspective of a peer reviewer, a change would often be a cause for concern. However, it is possible to design complex trials that anticipate the addition or subtraction of interventions, changes to the trial population, or are able to adapt or change during the course of the study. The Clinical Trials Facilitation and Coordination Group defines a complex clinical trial as a trial that has ‘separate parts that could constitute individual clinical trials and/or is characterised by prospective adaptions such as planned additions of new investigational medicinal products (IMPs) or trial populations’. Thus a complex clinical trial is often characterised by an overarching master protocol, describing a unified governance infrastructure, shared operational structures and practices, and common policies and procedures, creating a platform for a series of trials or intervention comparisons that are described in individual sub-protocols or appendices to the master protocol (Clinical Trials Faciitation and Coordination Group 2019). In this way, complex clinical trials using a master protocol design are often referred to as platform trials (Love et al. 2022; Noor et al. 2022) Fig. 1. However, in addition, there are several other broad designs of master protocol studies, and wide variation in the design of platform trials, which we have outlined below (Woodcock and LaVange 2017).

### Basket trials

Basket trials, or multi-cohort clinical trials, are a type of master protocol study that tests a single intervention in several groups of patients or populations (Fountzilas et al. 2022). This design is increasingly used in precision medicine where a particular targeted therapy is utilised in several distinct disease groups, most often cancer subtypes. For example, the B2225 trial evaluated tumour responses to imatinib (a monoclonal antibody) in patients with a variety of solid and haematological malignancies, and the BRAF V600 trial evaluated the response to vemurafenib in patients with a variety of non-melanoma cancers with the BRAF V600 mutation (Heinrich et al. 2008; Hyman et al. 2015). While basket trials offer more flexibility, in terms of the population under investigation, compared to parallel group trials, there remains limited ability to make changes to the design during the course of the study Fig. 2.

### Umbrella trials

Umbrella trials use a master protocol design to assess multiple interventions in a single population or disease group. Similar to a multi-arm parallel group trial, an umbrella trial can compare multiple interventions to a single control group, or multiple interventions to multiple control groups, akin to running multiple parallel group trials within a single overarching trial (Honap et al. 2024). For example, the NCI-MATCH trial evaluated multiple targeted treatments in patients with a specific gene mutation causing solid tumours, lymphoma, myeloma or rare cancers (O'Dwyer et al. 2023). Umbrella trials offer a much more convenient option for testing multiple, well-defined treatments in a single group of patients compared to setting up and running multiple simultaneous parallel group or multi-arm trials. However, the umbrella design requires the investigators to define the target population at the start of the trial and does not offer flexibility to adapt to changing circumstances, over and above what is possible within the constraints of traditional parallel group trials Fig. 3.

## Platform trials

Platform trials are master protocol studies, which have the ability to add or remove interventions during the course of the trial and, which can continue for many years (Woodcock and LaVange 2017). This allows investigators to test multiple hypotheses in multiple disease areas, using a single research governance infrastructure (Saville and Berry 2016). Platform trials encompass a variety of designs, including multi-arm multi-stage trials, multi-factorial trials, adaptive trials and common screening platform designs (Woodcock and LaVange 2017).

### Multi-arm multi-stage platform trials

Multi-arm multi-stage (MAMS) designs compare multiple interventions to a common control group. Interim analyses facilitate adaptions to trial conduct, including adjustments to sample size or close an arm dependent on pre-defined rules. MAMS designs have two principal benefits. First, there is an efficiency in sample size with the use of a common control group, compared to multiple parallel group trials, since multiple control groups are not needed. This immediately reduces the cost and duration of each intervention comparison. Second, the ability to stop recruitment to arms that show little evidence of benefit (or harm) at interim analysis provides further sample size efficiency and the opportunity to increase the rate of recruitment to other arms. This further reduces the duration of patient recruitment (Noor et al. 2022). A recent trial registry review identified 62 clinical trials that reported using a MAMS design. The majority (> 81%) of these trials targeted infectious disease or cancer disease areas, with 89% evaluating a drug therapy (Noor et al. 2022). The STAMPEDE trial is an example of a MAMS platform trial evaluating treatments for prostate cancer, which recruited nearly 12,000 men between 2005 and 2023. It evaluated nine treatments including drug therapies and radiotherapy at over 100 sites in the UK and seven sites in Switzerland (Parmar et al. 2017). Using the data from the STAMPEDE trial, Park et al. estimated that for ten interventions, a traditional parallel group approach would increase the cumulative duration by 312% and increase costs by 58% compared to a multi-arm multi-stage platform approach, suggesting that platform trials are likely to be faster and more cost effective compared to multiple parallel group trials (Park et al. 2022).

### Multifactorial platform trials

Multifactorial platform trials simultaneously assess multiple interventions to treat or prevent a disease. Depending on the specific eligibility criteria and trial design, a single participant may be eligible to receive multiple interventions, which increases the efficiency of the sample size and reduces the duration of participant recruitment. Multifactorial platform trials can be structured as multiple parallel group comparisons/multi-arm comparisons within a single platform, encompassing a range of eligibility criteria (Woodcock and LaVange 2017). The Randomised Embedded Multifactorial Platform for Community-acquired Pneumonia (REMAP-CAP) trial evaluated a suite of interventions to treat intensive care unit patients with COVID-19 disease, including the monoclonal antibody therapies tocilizumab and sarilumab (Andrews et al. 2024). The trial included over 24,000 randomisations across over 14,000 recruited patients, evaluating over 66 interventions in 298 hospitals worldwide. The REMAP-CAP trial structured interventions into categories of treatments called ‘domains’. Patients were randomly allocated to a treatment within each domain (if eligible), which facilitated the simultaneous testing of multiple treatments within a single disease area. This approach realised substantial efficiencies in sample size, compared to a conventional parallel group trial model (REMAP-CAP 2024).

**Fig. 1:**
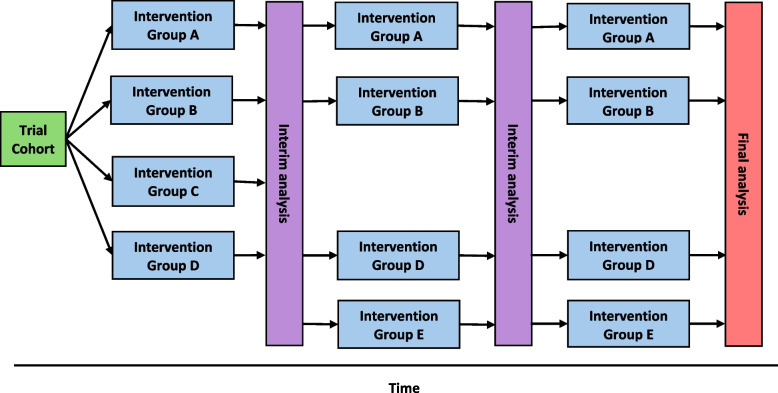
Basic structure of an adaptive platform trial with arms that can be added or removed throughout the course of the trial

**Fig. 2:**
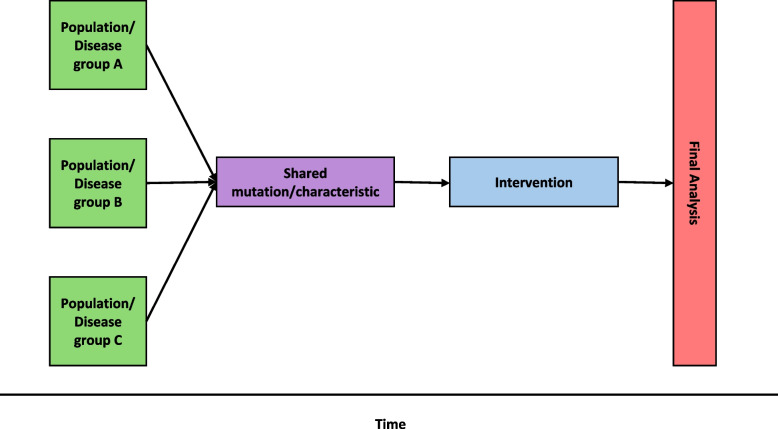
Basic structure of a basket trial

**Fig. 3:**
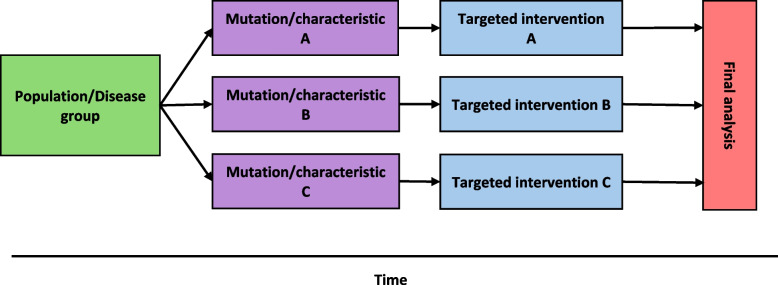
Basic structure of an umbrella trial

### Adaptive platform trials

The term ‘adaption’ or ‘adaptive’ is often used in the context of platform trials, but the meaning is sometimes unclear. The Adaptive Platform Trials Coalition provide consensus guidance, by delineating the scope of a master-protocol-based platform trial, from an adaptive trial, and describing areas of overlap—i.e. adaptive platform trials (Coalition APT 2019). As described above, a platform trial is a trial with a master protocol that can add or remove interventions. An adaptive trial is a trial that changes or adapts the trial conduct based on information generated from within and external to the trial, most often according to pre-specified rules, for example, dropping an interventional arm after interim analysis, based on pre-specified rules. Thus, an adaptive platform trial uses both a master protocol design and includes design features that enable the trial to respond or change within a defined set of parameters. This approach offers substantial flexibility in efficiently answering novel research questions.

### Trial within a cohort and common screening platform trials

The trial within a cohort (TWIC) design selects trial participants from within an existing cohort study (Bibby et al. 2018). This innovation is useful for trials of patients with rare diseases where identifying and screening patients can be challenging and sometimes prohibitive, or where there is infrastructure for an existing longitudinal cohort already established, which may promote efficient resource utilisation and cost savings for the trial component. TWICs are not typically prospectively planned from the perspective of the cohort study, but instead post hoc, utilising existing infrastructure. This approach generates several limitations. First, patients are often required to provide consent at multiple stages, for example at entry to the cohort and then at entry to any trial. Multi-stage consent may be a barrier to participation for some patient groups, particularly if there is a limitation of mental capacity. Second, the existing cohort governance apparatus is adapted to accommodate one or more trials. However, the governance structures may not be suitable. For example, data collection procedures that are satisfactory for a cohort study may not meet the regulatory requirements for a drug trial. An alternative model is a common screening platform design, where a master protocol is used to facilitate screening functions for an array of trials within a common disease area (Woodcock and LaVange 2017). For example, if there are several trials recruiting patients in a similar disease area, the same patient may be screened several times for multiple trials and/or recruited into one or more trials, where very similar data are collected. This duplicates a number of operational research tasks and places a significant burden of data collection repetition on the patient participant and the investigators. A common screening platform approach simplifies many screening and data collection tasks, sharing processes and data across multiple trials. While TWICs and common screening platforms are conceptually similar, they are operationally different, principally because a common screening platform is prospectively designed to facilitate clinical trials, while TWICs are not.

## Platform trial and master protocol design

The overall trial concept and protocol features common to all research questions or intervention comparisons are described in the master protocol, while individual research questions are described in sub-protocols or appendices to the master protocol, or as sub-sections within a single protocol. When a research question or intervention comparison is added to the platform, a new appendix or sub-protocol is added. The master protocol will include a statement that interventions can be added or removed during the life of the platform, which is different from a standard parallel group trial. The master protocol and appendices are structured so that the rules described in the master protocol apply to all research questions and all participants, but rules described in a protocol appendix only apply to that specific research question. Common features that might be described in the master protocol include: overall inclusion and exclusion criteria for entry to the platform; a common dataset or outcome set; consent procedures; randomisation procedures; data collection; pharmacovigilance and safety reporting; data sharing; dissemination; and governance structures. Each appendix to the master protocol will include, but not be limited to, a specific research question; additional details relating to the research question: specific inclusion and exclusion criteria; a description of the exposure/intervention; an outcome set; specific issues regarding intervention delivery; variations to the safety reporting procedures; and statistical methods including sample size and any pre-specified rules or adaptions to the analysis.

### Regulatory approvals and trial registration

The procedures for research ethics review, regulatory approval and trial registration will vary by jurisdiction. In the UK, the Medicine and Health Products Regulatory Authority (MHRA) is responsible for reviewing and approving clinical trial of investigational medicinal products (CTIMPs) and clinical trials of some medical devices. A platform trial that includes testing (or the capacity to test) drug interventions will need approval by the MHRA, which adds a layer of complexity if the platform will be used to predominantly test non-regulated interventions, which would not ordinarily come under the purview of the MHRA. Platform trials can be registered as single entities, with multiple research questions (protocol appendices) listed under a single clinical trial registration. Or, each research question can be registered as a separate clinical trial, linked by the trial title or master protocol (Clinical Trials Facilitation and Coordination Group 2019). Where a MAMS design is used or where one or more interventions will be compared to a common control group, it is advisable to register the platform as a single entity. In this case, the platform requires an overarching hypothesis, with each trial addressing a particular research question. Consideration of a platform trial with multiple independent research questions as a single clinical trial entity poses certain challenges from a research governance perspective under EU regulations [EU Clinical Trials Directive 2001/20/EC]. For example, how to apply the requirement for an end-of-trial report within 1 year of the end of trial, when individual research questions within a platform trial are operating independently (Clinical Trials Facilitation and Coordination Group 2019). Similarly, the timing of annual Development and Safety Update Reports (DSURs), which ordinarily would be on the anniversary of the initial clinical trial approval, may be difficult to synchronise with multiple independent research questions.

### Protocol amendments and risk assessment

When a new clinical trial is set up, the sponsor organisation will undertake a detailed governance review and risk assessment. During the course of the trial, if a substantial amendment to the protocol is requested, the sponsor will undertake a review of the proposed changes before submission to the ethics committee. However, this is not usually as detailed as the initial governance review. Bespoke processes may be needed for platform trials to ensure that thorough governance review and risk assessment is undertaken when new interventions or major adaptions are added. Similarly, it is entirely possible, and even desirable, that a platform trial should run for many years—much longer than a traditional clinical trial—which provides numerous benefits and efficiencies. However, this presents a fairly unusual situation whereby a participant could be enrolled in a clinical trial that has been open for, perhaps, a decade with policies and procedures that may be out of date by contemporary standards. The traditional clinical trial model safeguards against this situation by setting up a new clinical trial governance structure and undergoing a full governance review for each intervention. To ensure that policies and procedures for platform trials remain consistent with current guidelines and regulations, it is recommended that risk assessment and quality assurance reviews are undertaken on a regular basis.

### Statistical considerations

Platform trials offer substantial flexibility and adaptability in terms of the statistical design, which will vary according to the specific structure of each platform. A platform designed as a master protocol with multiple independent research questions, not involving a common control group, can be considered independent trials with independent statistical design and analysis approaches. This offers flexibility to use a variety of frequentist or Bayesian approaches, which could be different for each research question. A platform using a common control group, using a multi-arm multi-stage design or a pre-specified adaptive design, may have more limited statistical options and raises issues such as multiplicity and the use of concurrent vs non-concurrent controls in analysis. The FDA guidance on master protocol trials recommends including details of the statistical approach, which may include modifications to the sample size calculation, changes to the interventions, use of Bayesian methods or other adaptive strategies in the Statistical Analysis Plan (Food and Drug Administration 2022).

## Opportunities for platform trials

### Perioperative medicine and anaesthesia

Platform trials present a sentinel opportunity to make improvements to research delivery for anaesthesia and perioperative medicine, to enhance and expedite the benefits of clinical research. The surgical population is very large, which means that an established research platform has the scope to enrol a large number of participants and answer a research question in a short space of time compared to other disease groups (Abbott et al. 2017). Momentum is currently interrupted by the framework for conducting clinical trials and the limitations of setting up new research infrastructures for each research question. Perioperative care largely follows a stereotyped pathway across the majority of surgical specialties, involving multiple (predominantly non-evidence-based) clinical interventions and processes of care. Testing new interventions within this pathway is ideally suited to a platform trial approach, where changes to processes of care (e.g. induction of anaesthesia, surgical technique or devices, postoperative care etc.) can be tested within a single platform infrastructure. This could be enhanced by incorporating routinely collected Health Systems data (e.g. national Hospital Episode Statistics) and routinely collected electronic health record data to reduce the burden of research data collection and integrate clinical trials into routine clinical care.

The platform approach also offers an opportunity to improve participant diversity within trial samples, through two mechanisms. Firstly, the benefits of increased efficiency of research delivery will afford investigators proportionally more time to invest in identifying and recruiting underserved populations. Through unconscious bias, some groups within society can be missed or considered lower priority than other patient groups that are more commonly included in clinical trials. Secondly, due to the longevity of platform trials, which are designed to run for many years, it is more cost effective to invest in infrastructure or procedures to improve inclusivity, which may be overlooked or de-prioritised in traditional parallel group trials with a shorter operational duration. For example, fully embedded patient and public involvement activities, community engagement initiatives, translation of consent materials, and targeted interventions or procedures to address perceived barriers to inclusion. While many of these items should be addressed within the traditional clinical trial model, many are often overlooked due to funding or time constraints within a single project timeframe, which may be more feasible to address within a multi-year platform.

The use of platform trials in perioperative research is currently limited. However, there are several platforms under development in the UK and internationally. Examples include the following: the PROTECT platform led by researchers in the UK (protectresearch.org), the ANDES platform led by researchers in China (NCT06452147) and the Neo-VIKTORY platform (NCT06630130) led by investigators in South Korea. There are likely to be other platforms under development internationally, and we expect the numbers of operational platforms to increase over the next five years.

### Surgery

Surgery is well suited to platform trials; innovation is common, variation in practice is high, interventions may have heterogeneous treatment effects in different body compartments, and patients are at a high risk of adverse events; 1 in 2 patients develops a complication after major abdominal surgery for example. The first example of a platform trial in surgery was the ROSSINI-2 trial (ISRCTN78305317) funded by the NIHR in the UK, which is evaluating five different methods of postoperative infection prevention. It paved the way for further investment in efficient surgical research systems. ROSSINI-Platform will launch in 2025 as a factorial ‘Basket-MAMS’ design. It will build and expand ROSSINI-2 to test multiple interventions across six different surgical specialities: breast; vascular groin; cardiac; neurosurgery; leg amputations and obstetric surgery. Each of these surgical areas will form a'pillar'in the platform with a single overarching design structure, bringing efficiency in management, governance and conduct and allowing information of treatment effect estimates to be shared between pillars, informed by emerging data. The nature of surgical training is such that some elements of surgical technique remain dogmatic and hard to change (Arroyo et al. 2021; Bisset et al. 2024). However, increasing understanding of both segmentation and description of individual operation components has increased the feasibility and transparency of technical trials in surgery (Blencowe et al. 2016; El-Sayed et al. 2024). Elements of surgical care which involve both surgeons and the wider healthcare team also provide an exciting opportunity for collaboration. Perioperative Multifactorial Adaptive (PUMA) platform trial will test multiple interventions throughout the surgical care pathway as a patient moves through phases from consideration of a patient for surgery through to rehabilitation. It will permit both cluster and individual participation randomised interventions reflecting the complexity of some pathway changes with a high risk of crossover (e.g. a preoperative optimisation checklist). It will evaluate multiple areas of real-world practice variation to rapidly improve the evidence base for surgical care. Working together, surgeons and anaesthetists leading platform trials in similar disease areas, but different geographical areas presents an opportunity for federated data collection across a collaborative, agile, adaptive platform ecosystem (Glasbey et al. 2024).

### Low- and middle-income countries

There are several challenges facing surgical and perioperative systems in low- and middle-income countries (LMICs) (Collaborative G 2016; Collaborative G 2018; Surgery GCaNIfHRGHRUoG 2021). Most policy action focuses on improving capacity to deliver surgery (e.g. through National Surgical Obstetric and Anaesthesia Plans (NSOAPs)), neglecting the importance of research to improve surgical safety. Patients undergoing surgery in LMICs are two to three times more likely to develop a complication or die than in a high-income country (Surgery GCaNIfHRGHRUoG 2021; Biccard et al. 2018). Complications of surgery prevent patients’ return to normal life, including work, care and wider community responsibilities and increase the risk of financial catastrophe (Meara et al. 2015; Myles et al. 2022). It is estimated that postoperative death is comparable to the third leading cause of mortality worldwide and greater than HIV, TB and malaria combined; surgical complications are likely to be in the top five global causes of disability, although this remains ill-defined (Nepogodiev et al. 2019). Traditional research models are too slow, inflexible and expensive to rapidly deliver the evidence required to address this. Whilst innovative, efficient trial designs may hold the greatest benefit for groups at highest risk of adverse health outcomes, there remains limited experience in the Global South. *The Lancet*Series on Clinical Trials in Global Health identified less than ten master (i.e. platform) protocols registered in LMICs, and none of which included surgical patients (Park et al. 2021). The authors identified adaptive trials as the highest priority area for global health research, as they can sustain research infrastructure and human resources for long-term gains (Park et al. 2021; Chao et al. 2014; Alkire et al. 2015). One such trial is the MARLIN trial (NCT06465901) which is a multi-arm, multi-stage adaptive platform evaluating preoperative, intra-operative and postoperative strategies to prevent surgical wound infections in seven countries across three continents, led by surgeons in Benin (Lawani) and Rwanda (Ntirenganya). This will both test the feasibility of a platform design in surgery in low-resource settings and rapidly provide generalisable, contextually relevant clinical and cost effectiveness estimates to inform policy.

## Conclusions

The traditional model of clinical trials is increasingly recognised as time-consuming, inefficient and expensive. Platform trials represent a new paradigm for testing new treatments. Research questions in anaesthesia, surgery and perioperative medicine are well suited to a platform methodology.

## Data Availability

No datasets were generated or analysed during the current study.
